# Functional Effects of Toll-Like Receptor (TLR)3, 7, 9, RIG-I and MDA-5 Stimulation in Nasal Epithelial Cells

**DOI:** 10.1371/journal.pone.0098239

**Published:** 2014-06-02

**Authors:** Lotta Tengroth, Camilla Rydberg Millrud, Anne Månsson Kvarnhammar, Susanna Kumlien Georén, Leith Latif, Lars-Olaf Cardell

**Affiliations:** 1 Division of ENT Diseases, Department of Clinical Sciences, Intervention and Technology, Karolinska Institutet, Stockholm, Sweden; 2 Department of Otorhinolaryngology, Skåne University Hospital, Lund University, Malmö, Sweden; Centre d'Immunologie de Marseille-Luminy, CNRS-Inserm, France

## Abstract

**Background:**

The human nasal epithelium is an important physical barrier, and a part of the innate immune defense that protect against pathogens. The epithelial cells recognize microbial components by pattern-recognition receptors (PRRs), and thereby trigger an immune response. Even though TLR3, TLR7, TLR9, RIG-I and MDA-5 are all known to respond to viral stimulation, their potential role in chronic airway inflammation triggered by local cytokine release remains to be established.

**Methods:**

mRNA and corresponding protein expression of TLR3, TLR7, TLR9, RIG-I and MDA-5 were analyzed in nasal biopsies and various upper airway epithelial cell lines using real-time reverse transcription PCR, immunohistochemistry and flow cytometry. Ligand induced, cytokine release, was evaluated with ELISA.

**Results:**

Nasal biopsies were found to express TLR3, TLR7, TLR9, RIG-I and MDA-5, with the most abundant expression in the surface epithelium. These receptors were verified in primary human nasal epithelial cell (HNEC) as well as in the airway epithelial cell lines Detroit-562 and FaDu. Poly(I:C) (TLR3) and R-837 (TLR7) stimulation increased secretion of IL-6 and GM-CSF from the nasal mucosa and the epithelial cell lines. CpG (TLR9) stimulation caused release of IL-8 in the nasal mucosa and in FaDu. Poly(I:C)/LyoVec (RIG-I/MDA-5) stimulation activated the secretion of IFN-β in the nasal mucosa. A corresponding release was also detected from HNEC and Detroit-562.

**Conclusion:**

The nasal epithelium has the ability to recognize viral intrusion through TLR and RLR receptors, and the subsequent response might have a role in exacerbation of inflammatory diseases like allergic rhinitis and chronic rhinosinusitis.

## Introduction

The airway epithelium provides protection against pathogens [1], [2]. In addition to its barrier function, it is a major source of cytokines, chemokines, and other inflammatory mediators that affects both the adaptive and innate immune responses. Epithelial cells recognize conserved molecular motifs of microbial origin called pathogen-associated molecular patterns (PAMPs) by use of different pattern-recognition receptors (PRRs) [3]. PRRs, including Toll-like receptors (TLRs), nucleotide-binding oligomerization domain-like receptors (NLRs) and the recently discovered retinoic acid-inducible gene 1 (RIG-I)-like receptors (RLRs), are all known to play important roles in pathogen recognition, cell activation and regulation of immune responses [3], [4], [5]. Despite the protective function of PRRs against infections, accumulating evidence suggests a role for these receptors in the pathogenesis of various inflammatory diseases.

Mammals express at least 10 different TLRs that recognize components of bacteria and viruses, and they have been identified in several tissues and cells within the human airway [6], [7] The virus-recognizing TLRs, namely TLR3, TLR7, TLR8 and TLR9, respond to double-stranded (ds) RNA, single-stranded (ss) RNA and CpG-DNA, respectively [8], [9], [10]. The most recently discovered PRR members are the RLRs, comprising three homologues: RIG-I, melanoma differentiation-associated gene 5 (MDA-5), and laboratory of genetics and physiology 2 (LGP-2) [11]. RIG-I and MDA-5 detect RNA from replicating viruses in infected cells, which leads to the induction of type I interferons (IFNs) through the activation of the IFN regulating factor 3, and the production of proinflammatory cytokines by the activation of the nuclear factor (NF)-κB signaling pathway [12]. It has recently been shown that RIG-I is responsible for sensing viral RNA bearing triphosphate, while MDA-5 functions as a dsRNA sensor [13].

TLRs play important roles in host defense, but also contribute to the pathogenesis of specific diseases. Evidence suggests that there are intrinsic or locally induced deficiencies in epithelial barrier function of the nasal mucosa in patients with allergic rhinitis, due to persistent inflammation [14]. This inflammation is characterized by increased release of cytokine such as GM-CSF, infiltration of inflammatory cells and up-regulation of intercellular adhesion molecule-1 (ICAM-1) [15]. Defects in the host response to external pathogens, including viruses, have also been suggested to underlie the persistence of the inflammatory state [16]. Clinically, respiratory viral infections are also often implicated as triggers of flare-ups in patients with chronic rhinosinusitis (CRS) and these infections are also known to damage the function of human nasal epithelial cells (HNEC) [17], [18]. Several studies have shown abnormalities in the immune responses in patients with CRS, such as an exaggerated response to TLR3 [19]. dsRNA is known to bind to TLR3 and stimulate the expression of IL-8 in airway epithelial cells [20]. However, the role of all virus-recognizing PRRs on nasal epithelial cells has not yet been established. The aim of the present study was to characterize the expression and explore the activation of virus-recognizing PRRs on nasal epithelial cells as well as their functional response in the nasal mucosa. To this end, the nasal biopsies, primary human nasal epithelial cells and two complementary nasopharyngeal epithelial cells were used.

## Materials and Methods

### Ethics Statement

The study was approved by the Ethics Committees of Karolinska Institutet, Stockholm, Sweden. All participants gave their written informed consent, while all procedures were conducted according to the principles expressed in the Declaration of Helsinki.

### Sample collection

29 nasal biopsies were obtained from the inferior turbinate of healthy, non-smoking, volunteers (14 male, 15 female, ages 18–31). They were all symptom-free, with no history of allergic rhinitis and a negative skin prick test to the standard panel of allergens, including pollen, house dust mites, moulds and animal allergens (ALK Abelló, Hørsholm, Denmark). None of the participants in this study had any history of upper airway infection within 2 weeks before the time of visit, and they were all free of medication. The biopsies (approximately 2×2×2 mm in size) were sampled after topical application of local anesthesia containing lidocaine hydrochloride: nafazoline (34 mg/ml: 0.17 mg/ml), as previously described [9].

### Isolation of HNECs and cell lines

HNEC were obtained by nasal brushings of 13 healthy, non-smoking, volunteers (5 males, 8 females, age 26–51) as described by O'Brien et al. [21]. Cells were maintained in collagen-coated tissue culture flasks in complete medium containing keratinocyte serum-free medium (KSFM) (Invitrogen, Paisley, UK) supplemented with 0.05 mg/ml bovine pituitary extract, 5 ng/ml epidermal growth factor, 100 U/ml penicillin and 100 µg/ml streptomycin (Gibco, NY, USA). In the experiments cells from passages 2–5 were used and they were all positive for EpCAM (>90%), an adhesion molecule specific for epithelial cells [22].

Human pharyngeal epithelial cell lines Detroit-562 (CCL-138) and FaDu (HTB-43) (ATCC, Manassa, USA) was cultured in complete medium containing minimum essential medium (MEM) with Earl's salts and 2 mM L-glutamine (Gibco), supplemented with 10 % FBS (PAN Biotech, Aidenbach, Germany), 100 U/ml penicillin and 100 µg/ml streptomycin (Gibco). The medium for Detroit-562 also contained 1 mM sodium pyruvate (Sigma-Aldrich, St. Louis, USA), 0.1 mM non-essential amino acids, 50 µg/ml gentamicin and 0.25 µg/ml fungizone (Gibco). All cells were cultured at 37°C in a humidified 5% CO_2_ air atmosphere.

### RNA extraction and real-time reverse transcription-PCR

The biopsies and the epithelial cells were lysed in RLT buffer (Qiagen, Hilden, Germany) with 1% 2-mercaptoethanol. Total RNA was extracted using RNeasy Mini Kit (Qiagen), and the quantity and quality of the RNA were measured by spectrophotometry using the wavelength absorption ratio (260/280 nm). Reverse transcription of total RNA into cDNA was performed using the Omniscript reverse transcriptase kit (Qiagen) with oligo(dT)16 (DNA Technology, Aarhus, Denmark) in a Mastercycler personal PCR machine (Eppendorf, Hamburg, Germany). The RNA samples were denatured (65° for 5 min) and chilled (4° for 5 min). The reaction was carried out at 37°C for 1 h in a final volume of 20 µl.

Real-time reverse transcription PCR was performed using Stratagene Brilliant QPCR Mastermix (Agilent Technologies, Santa Clara, USA) and FAM dye-labelled probes for TLR3 (Hs00152933_ml), TLR7 (Hs00152971_ml), TLR9 (Hs00152973_ml), RIG-I (Hs01058986_m1), MDA-5 (Hs00223420_m1) and β-actin (Hs99999903_m1) (Applied Biosystems, Foster City, USA). The thermal cycler was set to perform an initial set-up (95°C for 10 min) and 45 cycles of denaturation (95°C for 30 s) followed by annealing/extension (60°C for 1 min) using a Stratagene Mx3000P (Agilent Technologies). The mRNA expression was assessed using the comparative cycle threshold (Ct) method where the relative amounts of mRNA were determined by subtracting Ct values of the detected gene with the Ct values of the housekeeping gene β-actin (ΔCt). The amount of mRNA is expressed in relation to 10^5^ β-actinmolecules, as 10^5^× 2^−ΔCt^ [23].

### Immunohistochemistry

The immunohistochemical staining was performed on paraffin embedded sections from 4 nasal biopsies (3 male, 1 female) and cultured HNEC, Detroit-562 and FaDu. The immunohistochemical staining was performed according to the labelled streptavidin biotin (LSAB^+^) System-horseradish peroxidase (HRP) kit (Dako, Copenhagen, Denmark), according to the manufacturer's instructions. Briefly, the cultured cells were seeded (300 000 cells/chamber) on 4-well Lab Tek chamber slides (Nalge Nunc International, Rochester, NY, USA) and grown to 80-90% confluence in complete medium. After three washes in PBS, cells were fixed in 4% formaldehyde. Slides were then rehydrated in PBS and treated with 0.05% hydrogen peroxide to quench endogenous peroxidase activity. The nasal biopsy sections were incubated at 4°C overnight and the epithelial cells at room temperature (RT) for 1 hour with monoclonal mouse anti-human Abs against TLR3 (Cat. no. 40C1285.6; AMS Biotechnology, Abingdon, UK), diluted 1∶20, TLR9 (211-MG-1TLR9; Acris antibodies, Hiddenhausen, Germany), diluted 1∶40 and RIG-I (ab77010; Abcam, Cambridge, UK), diluted 1∶20, or polyclonal rabbit anti-human Abs against TLR7 (ab45371), diluted 1∶20, and MDA-5 (ab69983; Abcam), diluted 1∶40. Counterstaining was performed with Mayer's haematoxylin. Thereafter, the glass slides were mounted in Faramount Aqueous Mounting Medium (Dako). As negative controls, N-series Universal Negative Control Reagents against mouse and rabbit (Dako) were utilized. Tris-buffered saline (TBS) (pH 7.6) supplemented with 0.05% Tween 20 (Sigma-Aldrich) was used for all washing steps. The sections were examined using light microscopy.

### FACS analysis

The epithelial cells were analysed on a Coulter Epics XL flow cytometer (Beckman Coulter, Marseille, France) and gated based on forward and side scatter properties. Events in the range 20 000–40 000 were collected and the data were analysed with the expo32 analysis software (Beckman Coulter). To identify intracellular protein expression, the IntraPrep permeabilization reagent kit (Beckman Coulter) was used according to the manufacturer's instructions. Briefly, cells were incubated with Abs for 20 min at RT, and thereafter washed and resuspended in phosphate-buffered saline (PBS). The conjugated mouse antibodies TLR3-FITC (clone 40C1285.6) and TLR9-PE (26C593.2) and rabbit polyclonal antibody TLR7-FITC was obtained from Imgenex (San Diego, CA, USA). Unlabeled mouse polyclonal antibodies against RIG-I (clone not specified; Abcam) were detected with the Alexa Fluor 488 msIgG2b labeling kit (Invitrogen, Carlsbad, CA, USA), whereas the unlabeled rabbit polyclonal antibodies against MDA-5 (Abcam) were detected with the labeled secondary antibody; goat polyclonal to rabbit IgG-FITC from AbD Serotec (Oxfordshire, UK). Isotype controls relevant for each antibody were used for background staining. The independent experiments were performed three times in singlets.

### Stimulation of nasal mucosa

Biopsies used for stimulation were collected in sterile, cold, complete medium containing KSFM supplemented with 0.05 mg/ml bovine pituitary extract, 5 ng/ml epidermal growth factor, 100 U/ml penicillin and 100 µg/ml streptomycin. The biopsies were separated into equally small pieces of 0.05 g and incubated in 1 ml of complete medium on 24-well culture plates at 37°C in a humidified 5% CO2 air atmosphere. The biopsies were stimulated in the absence (Untreated) or presence of 10 µg/ml poly(I:C), 10 µg/ml R-837, 1 µM phosphorothioate-modified CpG oligodeoxynucleotide 2006, 5′-tcgtcgttttgtcgttttgtcgtt-3′, (DNA Technology A/S), 1 µg/ml poly(I:C)/LyoVec (Invivogen, San Diego, CA, USA), or 10 ng/ml recombinant human tumor necrosis factor (TNF)-α (R&D Systems, Minneapolis, USA) for 24 h.

### Epithelial cell stimulation

HNEC, Detroit-562 and FaDu were seeded on 24-well culture plates (250 000 cells/well) in 1 ml complete medium and incubated overnight. Cells were then cultured for additionally 24 h in 1 ml complete medium in the absence (Untreated) or presence of 10 µg/ml poly(I:C), 10 µg/ml R-837, 1 µM phosphorothioate-modified CpG oligodeoxynucleotide 2006, 5′-tcgtcgttttgtcgttttgtcgtt-3′, 1 µg/ml poly(I:C)/LyoVec, or 10 ng/ml recombinant human TNF-α. The independent experiments were performed 5 to 9 times, all in duplicates. Cell-free culture supernatants were analysed for levels of interleukin (IL)-6 (antigen sensitivity: 3.1-30 pg/ml), IL-8 (31.2–2000 pg/ml) and granulocyte macrophage colony-stimulating factor (GM-CSF; 1-64 pg/ml) using ELISA plates from R&D Systems, as well as IFN-β (12.5–2000 pg/ml) using ELISA plates from PBL Interferon Source (NJ, USA), all according to the manufacturer's instructions.

### Statistics

Statistical analysis was performed using Graphpad Prism 5.01 (San Diego, CA, USA). *n* equals the number of independent experiments or donors. Data were analyzed using one-way repeated measures analysis of variance together with Dunnett's post test (for comparisons of more than two data sets). Data are expressed as mean ± SEM. *P*-values <0.05 were considered statistically significant.

## Results

### mRNA and protein expression in nasal tissue

**Figure 1:**
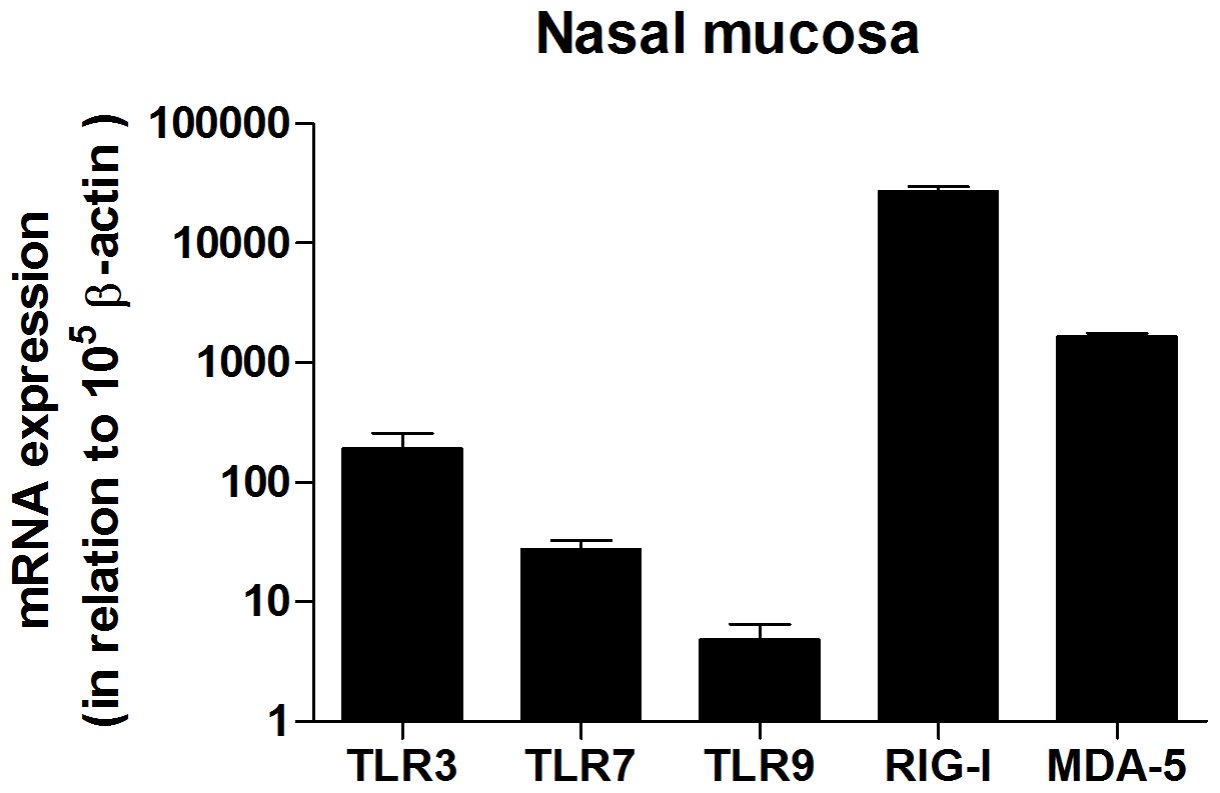
mRNA expression of TLR3, TLR7, TLR9, RIG-I and MDA-5 in human nasal mucosa. mRNA expression in nasal mucosal biopsies was determined by real-time reverse transcriptase-PCR (n = 20). Data is presented in relation to β-actin as 2-ΔCt × 105 and depicted in log scale as mean ± SEM.

**Figure 2:**
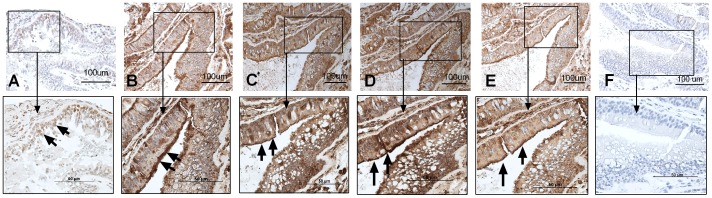
The nasal epithelium expresses TLR3, TLR7, TLR9, RIG-I and MDA-5. Sections of nasal biopsies were incubated with antibodies against TLR3 (**A**), TLR7 (**B**), TLR9 (**C**), RIG-I (**D**), and MDA-5 (**E**) and visualized by 3, 3′-diaminobenzidine (brown). In control slides (**F**), N-series universal negative control reagent was used. All sections were accompanied with a square magnification. All slides were counterstained with haematoxylin (blue). The figure shows one representative biopsy out of four (3 male, 1 female). The arrows indicate positive stained cells.

The presence of TLR3, TLR7, TLR9, RIG-I and MDA-5 in nasal mucosa biopsies was determined using real-time reverse transcriptase-PCR. Data showed significantly higher levels of RIG-I mRNA in the nasal mucosa, as compared to other PRRs measured. Expression of TLR3 and MDA-5 was also high, whereas TLR7 and TLR9 was more moderate (Fig. 1). Immunohistochemistry was used to determine the location of the receptor in the nasal mucosa. A strong expression for TLR7, TLR9, RIG-I and MDA-5 was seen, whereas TLR3 was moderately expressed. Overall, the staining was most abundant in the surface epithelium. It is also worth noting that a relatively strong staining of seromucous glands within the submucosa layer was also evident (Fig. 2A–E). Replacement of the specific primary antibodies with mouse isotype-matched controls (Fig. 2F) and rabbit isotype-matched controls resulted in a complete loss of staining (data not shown).

### TLR and RLR expression in epithelial cells

**Figure 3:**
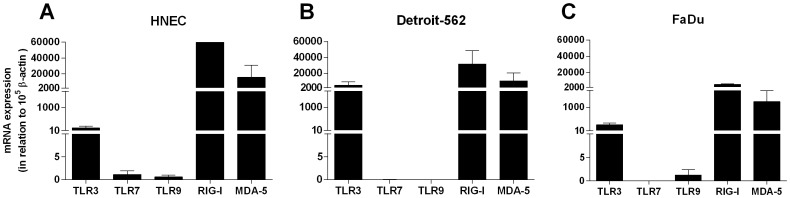
mRNA expression of TLR3, TLR7, TLR9, RIG-I and MDA-5 in epithelial cells. Levels of innate immune receptors in human nasal epithelial cells (HNEC) (n = 5) (A), Detroit-562 (n = 6) (B), and FaDu (n = 4) (C) was determined by real-time reverse transcriptase-PCR. Data is presented in relation to β-actin as 2-ΔCt × 105 and depicted in linear scale as mean ± SEM.

**Figure 4:**
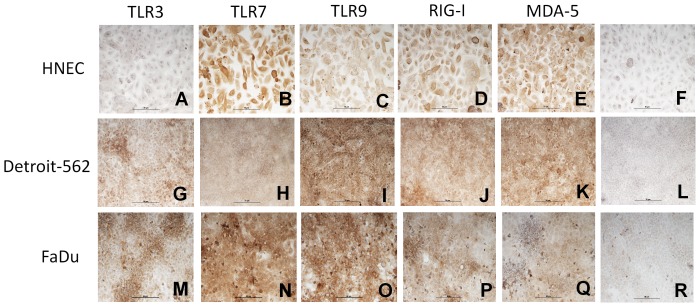
Expression of TLR3, TLR7, TLR9, RIG-I and MDA-5 on epithelial cells. Epithelial cells from primary HNEC (**A–E**), Detroit-562 (**G–K**) and FaDu (**M–Q**) were incubated with antibodies against TLR3, TLR7, TLR9, RIG-I, and MDA-5 and visualized by 3, 3′-diaminobenzidine (brown). In controls, N-series universal negative control reagent was used (**F, L, R**). All cells were counterstained with haematoxylin (blue). The figure shows one representative staining out of three independent experiments. The markers in the figure are 50 µm.

**Figure 5:**
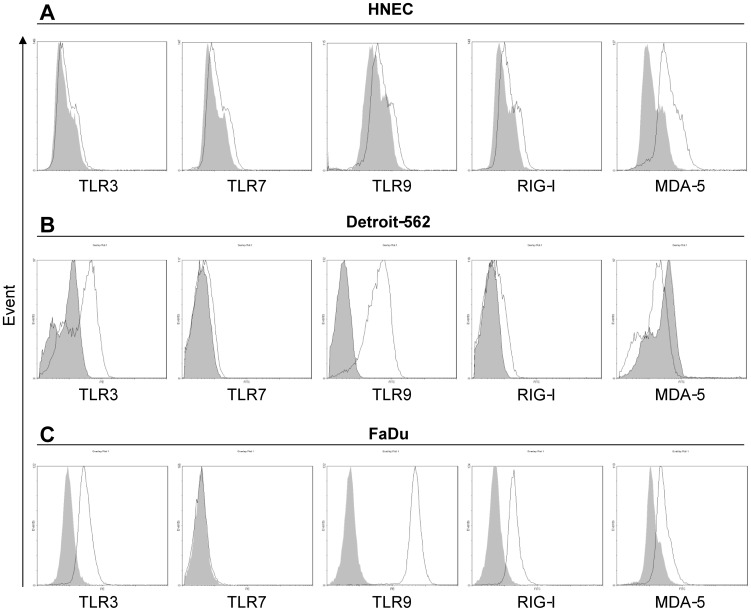
Expression of TLR3, TLR7, TLR9, RIG-I and MDA-5 proteins on epithelial cells. HNEC (**A**), Detroit-562 (**B**) and FaDu (**C**) were stained intracellularly with Abs against TLR3, TLR7, TLR9, RIG-I and MDA-5 (open histograms) or appropriate isotype control (shaded histograms) and analyzed by flow cytometry. Representative pictures from one out of three independent experiments are shown.

Since the staining of virus receptors was most abundant in the nasal epithelium, the expression of TLR3, TLR7, TLR9, RIG-I and MDA-5 was explored in primary HNEC and the nasopharyngeal epithelial cell lines Detroit-562 and FaDu using real-time reverse transcriptase-PCR, immunohistochemistry, and flow cytometry. mRNA data showed expression of all receptors in HNEC. Detroit-562 exhibited strong expression of TLR3, RIG-I and MDA-5, whereas the expression of TLR7 and TLR9 was barely detectable. FaDu exhibited significantly higher levels of RIG-I as compared to the other receptors measured. A strong expression of TLR3 and MDA-5 was also demonstrated, whereas the expression of TLR7 and TLR9 were barely detectable. (Fig. 3A–C). In contrast, all receptors could be demonstrated using immunohistochemistry. TLR3 was moderately expressed in HNEC (Fig. 4A), whereas a strong expression for TLR7, TLR9, RIG-I and MDA-5 was seen (Fig. 4B–E). Detroit-562 and FaDu expressed all receptors (Fig. 4G–K, M–Q). Replacement of the specific primary antibodies with mouse isotype-matched controls (Fig. 4F, L, R) and rabbit isotype-matched controls (data not shown), resulted in complete loss of staining. Using flow cytometry all receptors could be demonstrated in HNEC, Detroit-562 and FaDu with the exception of MDA-5 in Detriot-562 (Fig. 5A–C).

### In vitro stimulation of the nasal mucosa

**Figure 6:**
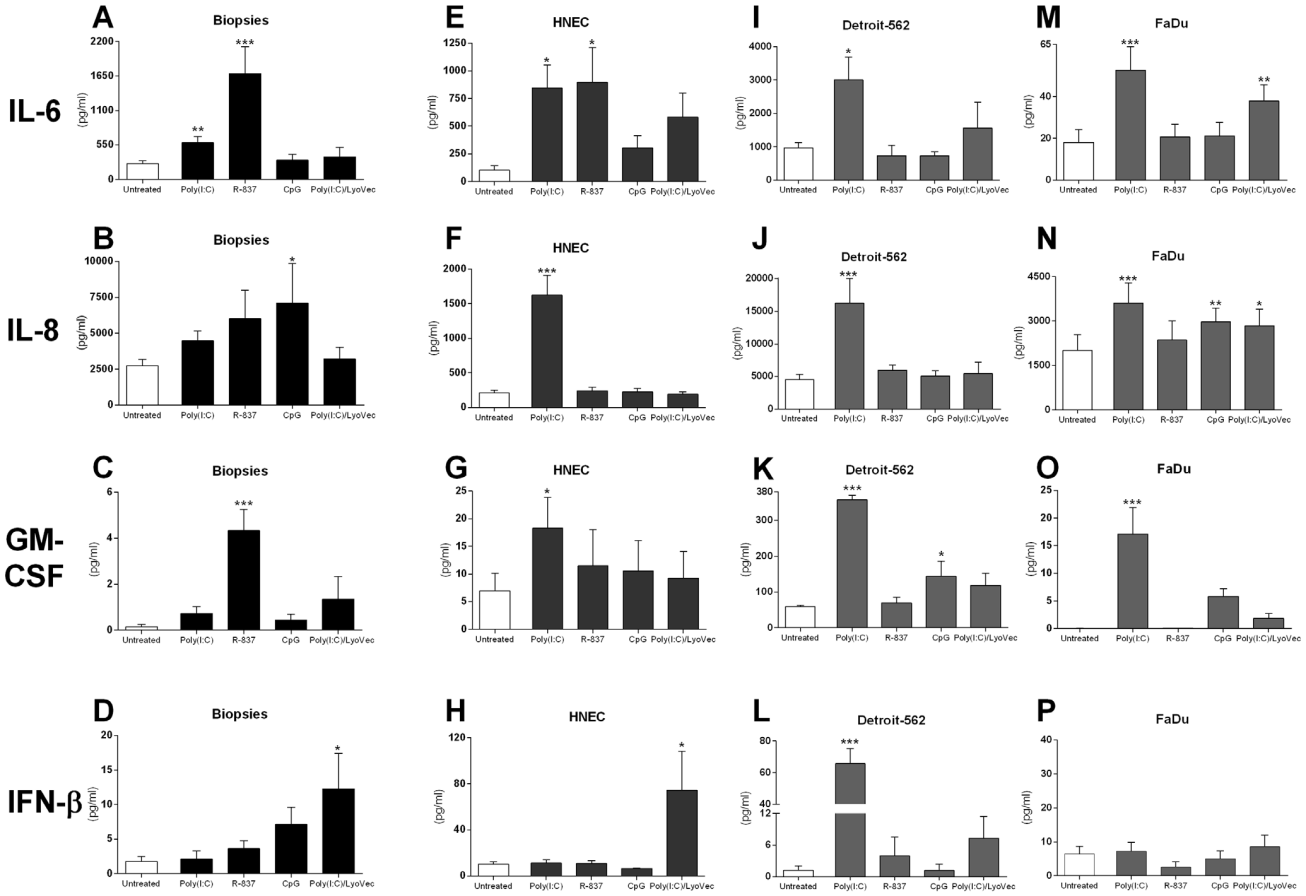
TLR3, TLR7, TLR9, RIG-I and MDA-5 stimulation promotes cytokine release. Nasal biopsies and epithelial cells were cultured in the absence (Untreated) or presence of 10 µg/ml poly(I:C) (TLR3), 10 µg/ml R-837 (TLR7), 1 µM CpG (TLR9) and 1 µg/ml poly(I:C)/LyoVec (RIG-I/MDA-5). TNF-α (10 ng/ml) was used as a positive control (data not shown). After 24 h, supernatants from nasal biopsies (n = 5) (**A–D**), HNEC (n = 6–9) (**E–H**), Detroit-562 (n = 5) (**I–L**) and FaDu (n = 5–9) (**M–P**) were collected and analyzed for levels of IL-6, IL-8, GM-CSF and IFN-β using ELISA. Data is presented as mean ± SEM of 5 to 9 independent experiments. *, p<0.05; **, p<0.01; ***, p<0.001.

TLR3, TLR7, TLR9, RIG-I and MDA-5 were found present in the nasal mucosa and their potential function upon ligand stimulation was therefore investigated. Biopsies were stimulated for 24 h with or without poly(I:C) (TLR3), R-837 (TLR7), CpG (TLR9) or poly(I:C)/LyoVec (RIG-I/MDA-5) followed by measurements of IL-6, IL-8, GM-CSF and IFN-β secretion. The cytokine release from ligand stimulated biopsies was compared to unstimulated biopsies (Untreated), in duplicates. TNF-α stimulated the secretion of all cytokines measured (data not shown). Stimulation with poly(I:C) resulted in an increased in IL-6, IL-8 and GM-CSF release (*P* = 0.04; *P* = 0.6; *P* = 0.9) although only IL-6 production reached statistical significance (Fig. 6A-C). R-837 stimulated a significant upregulation of IL-6 and GM-CSF production (*P*<0.01, *P*<0.01), whereas CpG stimulated a significant upregulation of IL-8 production (P = 0.04). Poly(I:C)/LyoVec stimulated a small increase in the release of IL-6 and GM-CSF, but a robust production of IFN-β was detected upon RIG-I/MDA-5 activation (P = 0.02) (Fig. 6D).

### Functional expression in cultured epithelial cells

In order to evaluate the receptor functionality on the epithelial cells, HNEC, Detroit-562 and FaDu were cultured for 24 h with or without poly(I:C) (TLR3), R-837 (TLR7), CpG (TLR9) or poly(I:C)/LyoVec (RIG-I/MDA-5) followed by measurements of IL-6, IL-8 and GM-CSF. The cytokine release from ligand stimulated cells was compared to unstimulated cells (Untreated), in duplicates. TNF-α stimulated the secretion of all cytokines measured (data not shown). In HNEC, stimulation with poly(I:C) gave rise to a significant production of IL-6, IL-8 and GM-CSF (*P* = 0.04; *P*<0.01; *P* = 0.04). R-837 gave rise to an increased production of IL-6 (*P* = 0.03) and GM-CSF (*P* = 0.6), all compared to cytokine production by untreated cells. Poly(I:C)/LyoVec also contributed to a small production of IL-6 and GM-CSF, although this did not reach statistical significance (Fig. 6E–G). In Detroit-562, stimulation with poly(I:C) caused a significant increase in the secretion of all cytokines (IL-6, *P* = 0.03; IL-8, *P*<0.01; GM-CSF, *P*<0.01), whereas CpG only induced a significant release of GM-CSF (*P* = 0.04). Poly(I:C)/LyoVec also stimulated an increase in IL-6 and GM-CSF secretion, but this increase did not reach statistical significance. No effects were seen with R-837 (Fig. 6I–K). In FaDu, Poly(I:C) and poly(I:C)/LyoVec also significantly increased the secretion of IL-6 (*P* = 0.01; *P* = 0.01). All ligands except R-837 significantly enhanced the release of IL-8 (Poly(I:C), *P* = 0.01; CpG, *P* = 0.01; Poly(I:C)/LyoVec, *P* = 0.01). GM-CSF levels were significantly increased following poly(I:C) stimulation (*P* = 0.01) and slightly enhanced after incubation with CpG and poly(I:C)/LyoVec (Fig. 6M–O).

HNEC, Detroit-562 and FaDu was also analysed for their ability to produce IFN-β upon TLR and RLR stimulation. Poly(I:C)/LyoVec increased the release of IFN-β in HNEC (P = 0.02) and Detroit-562 (*P* = 0.08), whereas no effects were seen on FaDu cells. Poly(I:C) also stimulated a significant release of IFN-β in Detroit-562 (P<0.01). No effects were seen upon stimulation of the remaining receptors (Fig 6H, L, P).

## Discussion

The presence of TLRs in airway tissues as well as on structural and immunocompetent cells is well documented [6], [24], [25], [26]. The present study extends the perspective of these findings by focusing on virus-recognizing PRRs in the nasal epithelium. We investigate the activation of the PRRs and demonstrate their specific role and function in the nasal mucosa. We initially noted that nasal biopsies expressed all receptors investigated. However, to fully understand the PRR expression in primary nasal epithelial cells, both mRNA and protein levels of the PRRs were analysed in isolated epithelial cells. The cell content in nasal biopsies primarily consists of nasal epithelial cells but also consists of neutrophils, leukocytes, goblet cells, brush border cells and endothelial cells lining the vessels. Therefore, through nasal brushing, we were able to collect chunks that consisted mainly of epithelial lining that were subsequently well preserved and thereby provided a culture of only HNEC for further analysis. Using this method, we found that TLR3, RIG-I and MDA-5 was expressed at both mRNA and protein levels in HNEC. These receptors were also expressed in all epithelial cell lines which is in line with a previous report by Broquet et al. stating a role for TLR3, RIG-I and MDA-5 in viral detection in the intestinal epithelium [27]. The discrepancy between mRNA and protein expression of TLR7 and TLR9 in Detroit-562 and FaDu can be explained by differences in posttranscriptional regulation, such as ubiquitination, phosphorylation, mRNA and protein turnover rates, or mRNA secondary structures that all have the ability to affect the protein translation efficiency [28]. It has been shown that mRNA has a shorter half-life compared to proteins in human cell lines, this could be an alternative explanation for the lower levels of the mRNA [29]. In combination, mRNA and protein levels provide us with a detailed understanding of the receptor's gene function [30].

The immunological activity of the airway epithelium involving cytokines, chemokines, and an array of other inflammatory mediators is essential for our host-defense against invading microbes [1]. However, there is always a risk for unwanted parallel effects of this defense system, especially if the patient is suffering from an ongoing chronic inflammatory disease [16]. In this study we directed our efforts on epithelial cell activity, induced by specific ligands of virus recognising PRRs in the nasal mucosa. IL-6, IL-8 and GM-CSF secretion were chosen as markers for epithelial cell activation, as they are powerful mediators of airway inflammation. IL-6 induces antibody production in B cells and T-cell activation and differentiation [31]. IL-8 is a major chemoattractant for neutrophils, and it stimulates these cells to release enzymes and produce reactive oxygen species [32]. Similarly, GM-CSF can prime both neutrophils and eosinophils for activation [33]. Several studies have also demonstrated that these cytokines are involved in virus induced exacerbations of inflammatory diseases [34], [35].

TLR3 was expressed in all our epithelial cell cultures and stimulation of the receptor activated a production of IL-6, IL-8 and GM-CSF from both the nasal mucosa as well as the epithelial cell lines. In addition to the ability of the TLR3 agonist to induce pro-inflammatory cytokines, it also promoted an upregulation of the viral epithelial cell receptor ICAM-1 (unpublished data). In line with our study, other reports have demonstrated that poly(I:C) induce the production of inflammatory cytokines/chemokine in nasal epithelial cells [36], as well as increased production of both IL-6 and IL-8 in human airway epithelial cells [37]. Rhinovirus (RV), a common dsRNA virus inducing cold in healthy individuals, is also recognized by TLR3 [38]. Studies have demonstrated elevated IL-6 and IL-8 production in nasal lavage fluids during common cold [39], [40]. This highlights the importance of epithelial TLR3, as a major mediator for the inflammatory and antiviral response in the nasal mucosa. TLR3 activation also causes unwanted parallel effects when the defense is activated [16]. Upregulation of TLR3 has been reported in the nasal mucosa of patients with allergic rhinitis [41]. Subauste et al. state that IL-6, IL-8 and GM-CSF produced by RV increase the susceptibility to further upper airway infection [42]. A recent study using a mouse model also suggested that TLR3 contributes to the exacerbation of virus-induced asthma [20]. This highlights the importance of TLR3 on epithelial cells, not only as a major mediator for the antiviral response, but also as an important player in virus-induced exacerbations of inflammatory diseases.

The present study demonstrates the presence of both TLR7 and TLR9 in the epithelial cell lines but only TLR7 stimulation upregulated the production of IL-6 and GM-CSF from the nasal mucosa. A similar cytokine production was also seen in epithelial cells, emphasizing the importance of TLR7 on epithelial cells in response to infections in the nasal mucosa. In line with this, Chehadeh et al. have demonstrated a TLR7 dependent IL-6 production after enterovirus infections [43]. This further emphasizes the role of TLR7 in viral recognition. Presently, CpG stimulation of the nasal mucosa significantly increased IL-8 secretion, whereas no such thing was seen in supernatants from the epithelial cells. Zhao et al. have reported that TLR9 is highly up-regulated in CRS patients with nasal polyps [44]. This indicates that also TLR9 activation could have an important role in exacerbation of inflammatory airway diseases like CRS.

RIG-I/MDA-5 activation resulted in upregulated IFN-β release in the nasal mucosa. A similar release was also seen in our epithelial cell lines, demonstrating that epithelial cells are responsible for the nasal mucosa release of IFN-β early upon viral infection. IFN-β is essential for the induction of a robust immune response in response to viral infections and previous studies have shown that epithelial cells pre-treated with IFN-β are protected against subsequent viral infections [45], even days after stimulation [46]. Wark et al. show that the impairment of virus-induced IFN-β expression is associated with enhanced viral replication in cell cultures [47]. Liu et al. has also suggested that IFN-β secreted from respiratory syncytial virus-infected epithelial cells induces TLR expression in a paracrine fashion in A549 cells [48]. This underscores the importance of RIG-I/MDA-5 on epithelial cells mediating an early antiviral response to viral infections in the nasal mucosa. RIG-I and MDA-5 have also been suggested to be involved in various inflammatory diseases, like asthma. Asthmatic patients are known to be more susceptible to RV infection due to impaired RIG-I/MDA-5 production of IFN-β [47], among the infected epithelial cells. In analogy, MDA-5−/− mice exhibit decreased IFN mRNA expression five days after a virus infection, resulting in increased mortality and severe histopathological changes in the lower airway [49]. Further, RV-infected, OVA-treated, MDA-5−/− mice have shown even more reduced IFN levels compared to wild-type mice. This suggests that virus infections via the activation of RIG-I/MDA-5 on epithelial cells could be involved in virus induced exacerbations of inflammatory diseases in the nasal mucosa.

In support of the present data, Matijevic et al, have reported that the same TLR agonist can cause disparate patterns of IL-6 production and cytokine regulation in Detroit-562 and FaDu cells [50], [51]. This could explain some of the differences in cytokine release presently seen between the cell lines tested and HNEC. The obtained differences could also be related to the diverse origin of the epithelial cell lines. FaDu cells originate from primary hypopharyngeal carcinoma, Detroit-562 cell line from a metastasis of pharynx carcinoma, whereas HNEC are isolated from the inferior turbinate of healthy individuals. Hence, HNEC is therefore probably the cell type that best reflects the nasal response to a viral attack.

To summarize, this study demonstrates that TLR3, TLR7, TLR9, RIG-I and MDA-5 are expressed on human nasal epithelial cells can and recognize virus-related products causing an increased inflammatory response. Such activates have previously not been reported for RIG-I and MDA-5. Especially the presented induction of IFN-β underscores that viruses via PRRs on epithelial cells can have the ability to affect an ongoing inflammatory processes in the nasal mucosa. Hence, it is tempting to speculate in a role for epithelial expressed-, virus-recognizing PRRs in exacerbations of inflammatory diseases like allergic rhinitis and CRS.

## References

[1] Holgate ST (2007) Epithelium dysfunction in asthma. Journal of Allergy and Clinical Immunology 120: 1233–1246. 18073119 10.1016/j.jaci.2007.10.025

[2] Schleimer RP, Kato A, Kern R, Kuperman D, Avila PC (2007) Epithelium: At the interface of innate and adaptive immune responses. Journal of Allergy and Clinical Immunology 120: 1279–1284. 17949801 10.1016/j.jaci.2007.08.046 PMC2810155

[3] Bals R, Hiemstra PS (2004) Innate immunity in the lung: how epithelial cells fight against respiratory pathogens. European Respiratory Journal 23: 327–333. 14979512 10.1183/09031936.03.00098803

[4] Kawai T, Akira S (2006) TLR signaling. Cell Death and Differentiation 13: 816–825. 16410796 10.1038/sj.cdd.4401850

[5] Takeuchi O, Akira S (2010) Pattern recognition receptors and inflammation. Cell 140: 805–820. 20303872 10.1016/j.cell.2010.01.022

[6] Sukkar MB, Xie SP, Khorasani NM, Kon OM, Stanbridge R, et al (2006) Toll-like receptor 2, 3, and 4 expression and function in human airway smooth muscle. Journal of Allergy and Clinical Immunology 118: 641–648. 16950283 10.1016/j.jaci.2006.05.013

[7] Janssens S, Beyaert R (2003) Role of toll-like receptors in pathogen recognition. Clinical Microbiology Reviews 16: : 637–+. 10.1128/CMR.16.4.637-646.2003 PMC207104 14557290

[8] Abreu MT, Arditi M (2004) Innate immunity and toll-like receptors: Clinical implications of basic science research. Journal of Pediatrics 144: 421–429. 15069387 10.1016/j.jpeds.2004.01.057

[9] Fransson M, Benson M, Erjefalt JS, Jansson L, Uddman R, et al (2007) Expression of Toll-like receptor 9 in nose, peripheral blood and bone marrow during symptomatic allergic rhinitis. Respir Res 8: 17. 17328813 10.1186/1465-9921-8-17 PMC1810251

[10] Akira S, Hemmi H (2003) Recognition of pathogen-associated molecular patterns by TLR family. Immunol Lett 85: 85–95. 12527213 10.1016/s0165-2478(02)00228-6

[11] Takahasi K, Kumeta H, Tsuduki N, Narita R, Shigemoto T, et al (2009) Solution Structures of Cytosolic RNA Sensor MDA5 and LGP2 C-terminal Domains IDENTIFICATION OF THE RNA RECOGNITION LOOP IN RIG-I-LIKE RECEPTORS. Journal of Biological Chemistry 284: 17465–17474. 19380577 10.1074/jbc.M109.007179 PMC2719387

[12] Li XJ, Ranjith-Kumar CT, Brooks MT, Dharmaiah S, Herr AB, et al (2009) The RIG-I-like Receptor LGP2 Recognizes the Termini of Double-stranded RNA. Journal of Biological Chemistry 284: 13881–13891. 19278996 10.1074/jbc.M900818200 PMC2679488

[13] Hartmann G (2007) Triphosphate-RNA is the ligand for the cytosolic receptor retinoic-acid inducible gene-1 (RIG-I). Inflammation Research 56: S328–S329.

[14] Tieu DD, Kern RC, Schleimer RP (2009) Alterations in epithelial barrier function and host defense responses in chronic rhinosinusitis. Journal of Allergy and Clinical Immunology 124: 37–42. 19560577 10.1016/j.jaci.2009.04.045 PMC2802265

[15] Canonica GW, Compalati E (2009) Minimal persistent inflammation in allergic rhinitis: implications for current treatment strategies. Clinical and Experimental Immunology 158: 260–271. 19765020 10.1111/j.1365-2249.2009.04017.x PMC2792821

[16] Kern RC, Conley DB, Walsh W, Chandra R, Kato A, et al (2008) Perspectives on the etiology of chronic rhinosinusitis: An immune barrier hypothesis. American Journal of Rhinology 22: 549–559. 18786300 10.2500/ajr.2008.22.3228 PMC2802263

[17] Gwaltney JM Jr (1996) Acute community-acquired sinusitis. Clin Infect Dis 23: : 1209–1223; quiz 1224–1205. 10.1093/clinids/23.6.1209 PMC7109987 8953061

[18] Pedersen M, Sakakura Y, Winther B, Brofeldt S, Mygind N (1983) Nasal mucociliary transport, number of ciliated cells, and beating pattern in naturally acquired common colds. Eur J Respir Dis Suppl 128 (Pt 1): 355–365. 6578083

[19] Hamilos DL (2014) Host-microbial interactions in patients with chronic rhinosinusitis. J Allergy Clin Immunol 133: : 640–653 e644. 10.1016/j.jaci.2013.06.049 PMC7112254 24290275

[20] Torres D, Dieudonne A, Ryffel B, Vilain E, Si-Tahar M, et al (2010) Double-Stranded RNA Exacerbates Pulmonary Allergic Reaction through TLR3: Implication of Airway Epithelium and Dendritic Cells. Journal of Immunology 185: 451–459. 10.4049/jimmunol.0902833 20505141

[21] O'Brien GJ, Riddell G, Elborn JS, Ennis M, Skibinski G (2006) Staphylococcus aureus enterotoxins induce IL-8 secretion by human nasal epithelial cells. Respir Res 7: 115. 16952309 10.1186/1465-9921-7-115 PMC1579218

[22] Balzar M, Winter MJ, de Boer CJ, Litvinov SV (1999) The biology of the 17-1A antigen (Ep-CAM). Journal of Molecular Medicine-Jmm 77: 699–712. 10.1007/s001099900038 10606205

[23] Petterson T, Mansson A, Riesbeck K, Cardell LO (2011) Nucleotide-binding and oligomerization domain-like receptors and retinoic acid inducible gene-like receptors in human tonsillar T lymphocytes. Immunology 133: 84–93. 21342182 10.1111/j.1365-2567.2011.03414.x PMC3088970

[24] Lesmeister MJ, Bothwell MR, Misfeldt ML (2006) Toll-like receptor expression in the human nasopharyngeal tonsil (adenoid) and palantine tonsils: A preliminary report. International Journal of Pediatric Otorhinolaryngology 70: 987–992. 16325925 10.1016/j.ijporl.2005.10.009

[25] Uehara A, Fujimoto Y, Fukase K, Takada H (2007) Various human epithelial cells express functional Toll-like receptors, NOD1 and NOD2 to produce anti-microbial peptides, but not proinflammatory cytokines. Molecular Immunology 44: 3100–3111. 17403538 10.1016/j.molimm.2007.02.007

[26] Bogefors J, Kvarnhammar AM, Latif L, Petterson T, Uddman R, et al (2011) Retinoic acid-inducible gene 1-like receptors in the upper respiratory tract. American Journal of Rhinology & Allergy 25: E262–E267. 22185736 10.2500/ajra.2011.25.3712

[27] Broquet AH, Hirata Y, McAllister CS, Kagnoff MF (2011) RIG-I/MDA5/MAVS Are Required To Signal a Protective IFN Response in Rotavirus-Infected Intestinal Epithelium. Journal of Immunology 186: 1618–1626. 10.4049/jimmunol.1002862 21187438

[28] Qian C, Cao XT (2013) Regulation of Toll-like receptor signaling pathways in innate immune responses. Translational Immunology in Asia-Oceania 1283: 67–74. 10.1111/j.1749-6632.2012.06786.x 23163321

[29] Laurent JM, Vogel C, Kwon T, Craig SA, Boutz DR, et al (2010) Protein abundances are more conserved than mRNA abundances across diverse taxa. Proteomics 10: 4209–4212. 21089048 10.1002/pmic.201000327 PMC3113407

[30] Fu N, Drinnenberg I, Kelso J, Wu JR, Paabo S, et al. (2007) Comparison of Protein and mRNA Expression Evolution in Humans and Chimpanzees. PLoS One 2.. 10.1371/journal.pone.0000216 PMC1789144 17299596

[31] Akira S, Hirano T, Taga T, Kishimoto T (1990) Biology of Multifunctional Cytokines - Il-6 and Related Molecules (Il-1 and Tnf). Faseb Journal 4: 2860–2867. 2199284

[32] Leonard EJ, Yoshimura T (1990) Neutrophil Attractant Activation Protein-1 (Nap-1 [Interleukin-8]). American Journal of Respiratory Cell and Molecular Biology 2: 479–486. 2189453 10.1165/ajrcmb/2.6.479

[33] Lopez AF, Williamson DJ, Gamble JR, Begley CG, Harlan JM, et al (1986) Recombinant Human Granulocyte-Macrophage Colony-Stimulating Factor Stimulates Invitro Mature Human Neutrophil and Eosinophil Function, Surface-Receptor Expression, and Survival. Journal of Clinical Investigation 78: 1220–1228. 3021817 10.1172/JCI112705 PMC423807

[34] Nicholson KG, Kent J, Ireland DC (1993) Respiratory Viruses and Exacerbations of Asthma in Adults. British Medical Journal 307: 982–986. 8241910 10.1136/bmj.307.6910.982 PMC1679193

[35] Pattemore PK, Johnston SL, Bardin PG (1992) Viruses as Precipitants of Asthma Symptoms.1. Epidemiology. Clinical and Experimental Allergy 22: 325–336. 1586873 10.1111/j.1365-2222.1992.tb03094.x PMC7162032

[36] Matsumoto M, Seya T (2008) TLR3: interferon induction by double-stranded RNA including poly(I:C). Adv Drug Deliv Rev 60: 805–812. 18262679 10.1016/j.addr.2007.11.005

[37] Golebski K, Luiten S, van Egmond D, de Groot E, Roschmann KI, et al (2014) High Degree of Overlap between Responses to a Virus and to the House Dust Mite Allergen in Airway Epithelial Cells. PLoS One 9: e87768. 24498371 10.1371/journal.pone.0087768 PMC3912021

[38] Wang Q, Nagarkar DR, Bowman ER, Schneider D, Gosangi B, et al (2009) Role of Double-Stranded RNA Pattern Recognition Receptors in Rhinovirus-Induced Airway Epithelial Cell Responses. Journal of Immunology 183: 6989–6997. 10.4049/jimmunol.0901386 PMC2920602 19890046

[39] Dassonville C, Bonfils P, Momas I, Seta N (2007) Nasal inflammation induced by a common cold: comparison between controls and patients with nasal polyposis under topical steroid therapy. Acta Otorhinolaryngol Ital 27: 78–82. 17608135 PMC2640005

[40] Fleming HE, Little FF, Schnurr D, Avila PC, Wong H, et al (1999) Rhinovirus-16 colds in healthy and in asthmatic subjects: similar changes in upper and lower airways. Am J Respir Crit Care Med 160: 100–108. 10390386 10.1164/ajrccm.160.1.9808074

[41] Fransson M, Adner M, Erjefalt J, Jansson L, Uddman R, et al.. (2005) Up-regulation of Toll-like receptors 2, 3 and 4 in allergic rhinitis. Respiratory Research 6. 10.1186/1465-9921-6-100 PMC1243240 16146574

[42] Subauste MC, Jacoby DB, Richards SM, Proud D (1995) Infection of a Human Respiratory Epithelial-Cell Line with Rhinovirus - Induction of Cytokine Release and Modulation of Susceptibility to Infection by Cytokine Exposure. Journal of Clinical Investigation 96: 549–557. 7615827 10.1172/JCI118067 PMC185229

[43] Chehadeh W, Alkhabbaz M (2013) Differential TLR7-mediated expression of proinflammatory and antiviral cytokines in response to laboratory and clinical enterovirus strains. Virus Research 174: 88–94. 23523654 10.1016/j.virusres.2013.03.006

[44] Zhao CY, Wang X, Liu M, Jin DJ (2011) Microarray Gene Analysis of Toll-Like Receptor Signaling Elements in Chronic Rhinosinusitis with Nasal Polyps. International Archives of Allergy and Immunology 156: 297–304. 21720175 10.1159/000323767

[45] Cakebread JA, Xu YH, Grainge C, Kehagia V, Howarth PH, et al (2011) Exogenous IFN-beta has antiviral and anti-inflammatory properties in primary bronchial epithelial cells from asthmatic subjects exposed to rhinovirus. Journal of Allergy and Clinical Immunology 127: 1148–U1416. 21329968 10.1016/j.jaci.2011.01.023

[46] Gaajetaan GR, Geelen TH, Vernooy JH, Dentener MA, Reynaert NL, et al (2013) Interferon-beta induces a long-lasting antiviral state in human respiratory epithelial cells. J Infect 66: 163–169. 23201152 10.1016/j.jinf.2012.11.008

[47] Wark PAB, Johnston SL, Bucchieri F, Powell R, Puddicombe S, et al (2005) Asthmatic bronchial epithelial cells have a deficient innate immune response to infection with rhinovirus. Journal of Experimental Medicine 201: 937–947. 15781584 10.1084/jem.20041901 PMC2213100

[48] Liu P, Jamaluddin M, Li K, Garofalo RP, Casola A, et al (2007) Retinoic acid-inducible gene I mediates early antiviral response and Toll-like receptor 3 expression in respiratory syncytial virus-infected airway epithelial cells. J Virol 81: 1401–1411. 17108032 10.1128/JVI.01740-06 PMC1797494

[49] Gitlin L, Benoit L, Song C, Cella M, Gilfillan S, et al. (2010) Melanoma Differentiation-Associated Gene 5 (MDA5) Is Involved in the Innate Immune Response to Paramyxoviridae Infection In Vivo. Plos Pathogens 6.. 10.1371/journal.ppat.1000734 PMC2809771 20107606

[50] Matijevic T, Marjanovic M, Pavelic J (2009) Functionally Active Toll-Like Receptor 3 on Human Primary and Metastatic Cancer Cells. Scandinavian Journal of Immunology 70: 18–24. 19522763 10.1111/j.1365-3083.2009.02262.x

[51] Matijevic T, Pavelic J (2011) The dual role of TLR3 in metastatic cell line. Clinical & Experimental Metastasis 28: 701–712. 21735101 10.1007/s10585-011-9402-z

